# Complete genome sequence of *Limosilactobacillus fermentum* strain VNUA2025

**DOI:** 10.1128/mra.01422-25

**Published:** 2026-04-20

**Authors:** Thuy Thi Thu Trinh, Son Truong Dinh, Hoan Van Duong, Canh Xuan Nguyen

**Affiliations:** 1 Faculty of Biotechnology, Vietnam National University of Agriculture 166265, Hanoi, Vietnam; University of Wisconsin-Madison 5228, Madison, Wisconsin, USA

**Keywords:** *Limosilactobacillus fermentum*, VNUA2025, probiotic

## Abstract

Here, we present the complete genome sequence of *Limosilactobacillus fermentum* strain VNUA2025, isolated from diseased fish samples in Vietnam. The genome was obtained by integrating sequencing data generated using Oxford Nanopore’s PromethION platform with an R10.4.1 flow cell. The fully assembled circular genome is 2,110,961 bp, with a GC content of 51.51%.

## ANNOUNCEMENT

*Limosilactobacillus fermentum* strain VNUA2025 was isolated from the intestines of grass carp collected in Hai Phong Province, Vietnam. Intestinal samples were either stored at 4°C prior to processing or immediately subjected to isolation using the serial dilution method (1).

The VNUA2025 strain was cultured in 20 mL of MRS medium at 30°C, with shaking at 200 rpm for 48 h. The culture was then centrifuged at 10,000g for 10 min to harvest the cells. Total genomic DNA was extracted using a standard phenol–chloroform extraction protocol followed by isopropanol precipitation (2). The concentration, integrity, and purity of the genomic DNA (gDNA) were then evaluated. The genomic DNA was sequenced on the Oxford Nanopore’s PromethION platform using an R10.4.1 flow cell.

The DNA library was prepared using the Native Barcoding Kit 24 V14 protocol (SQK-NBD114.24, Oxford Nanopore Technologies, UK) with slight modifications: the end-repair and ligation steps were extended to 30 min, and wide-bore pipette tips with gentle flicking were used to minimize DNA shearing. Raw FAST5 files were base-called with Guppy v6.4.6 in super-accuracy mode, generating a total of 930,898 reads, corresponding to approximately 8.37 Gb of raw data. The reads had a median length of 7.0 kb and a mean length of 9.0 kb, with an N50 of 12.8 kb. Mean and median quality scores were 21.4 and 21.3, respectively, and notably, 100% of reads exceeded Q20.

Base calling was performed using Guppy v6.4.6 in super-accurate mode. Reads shorter than 5,000 bp or with a Q score ≤20 were removed using Chopper v0.5 (3). *De novo* assembly of the filtered reads was performed using the Flye v2.9-b1768 using the parameters --nano-hq and --read-error 0.03 on Nanopore super-accuracy base-called data (4), yielding the complete circular genome of *L. fermentum* strain VNUA2025 of approximately 2.11 Mb with an average coverage of ~3,145×.

The completeness of the genome assembly was assessed using BUSCO v5.4.5 (5) in prok_genome mode, utilizing the bacteria_odb10 lineage data set (released on 8 January 2024). The VNUA2025 assembly showed >98.4% completeness. Default parameters were used for all software unless otherwise noted. These metrics indicate a high-quality, contiguous genome assembly, suitable for downstream genomic analyses.

The VNUA2025 genome showed an ANI score of 98.83% and an OrthoANI score of 98.88% when compared with the genome of *L. fermentum* IMDO 130101 (GCF_900205745.1) (6), thereby confirming its identification as *L. fermentum*. Genome annotation, including the identification of genes, proteins, and functional features, was performed using the RAST server (Rapid Annotation using Subsystem Technology; https://rast.nmpdr.org/) (7, 8), which identified 57 tRNAs and 15 rRNAs. Additionally, GeneMark (http://exon.gatech.edu/GeneMark/genemarks2.cgi) predicted 2,120 protein-coding genes.

These high-quality, contiguous genome assemblies are suitable for downstream genomic analyses.

## Data Availability

The whole-genome sequence and associated data have been deposited at GenBank (accession number CP197324) and the Sequence Read Archive (SRR33928577).
